# Gravitational stress during parabolic flights reduces the number of circulating innate and adaptive leukocyte subsets in human blood

**DOI:** 10.1371/journal.pone.0206272

**Published:** 2018-11-14

**Authors:** Ulrik Stervbo, Toralf Roch, Tina Kornprobst, Birgit Sawitzki, Gerald Grütz, Andreas Wilhelm, Francis Lacombe, Kaoutar Allou, Markus Kaymer, Antoine Pacheco, Jacques Vigne, Timm H. Westhoff, Felix S. Seibert, Nina Babel

**Affiliations:** 1 Center for Translational Medicine - Medical Clinic I, Marien Hospital Herne - University Hospital of the Ruhr-University Bochum, Herne, Germany; 2 Charité – Universitätsmedizin Berlin, corporate member of Freie Universität Berlin, Humboldt-Universität zu Berlin, and Berlin Institute of Health, Berlin, Germany; 3 Laboratoire d’hématologie, CHU de Bordeaux, Hôpital Haut-Lévêque, Pessac, France; 4 Beckman Coulter GmbH, Krefeld, Germany; 5 Beckman Coulter France S.A.S., Villepinte, France; Charles P. Darby Children’s Research Institute, UNITED STATES

## Abstract

Gravitational stress occurs during space flights or certain physical activities including extreme sports, where the change in experienced gravitational acceleration can reach large magnitudes. These changes include reduction and increase in the physical forces experienced by the body and may potentially induce pathogenic alterations of physiological processes. The immune system is known to regulate most functions in the human organism and previous studies suggest an impairment of the immune function under gravitational stress. However, systematic studies aiming to investigate the effect of gravitational stress on cellular immune response in humans are lacking. Since parabolic flights are considered as feasible model to investigate a short-term impact of gravitational changes, we evaluated the influence of gravitational stress on the immune system by analyzing leukocyte numbers before and after parabolic flight maneuvers in human blood. To correct for circadian effects, samples were taken at the corresponding time points on ground the day before the flight. The parabolic flight maneuvers led to changes in numbers of different leukocyte subsets. Naïve and memory T and B cell subsets decreased under gravitational stress and lower numbers of basophils and eosinophils were observed. Only circulating neutrophils increased during the parabolic flight. The observed changes could not be attributed to stress-induced cortisol effects, since cortisol levels were not affected. Our data demonstrate that the gravitational stress by parabolic flights can affect all parts of the human immune system. Consequently, it is possible that gravitational stress can have clinically relevant impacts on the control of immune responses.

## Introduction

All physiological processes including immunological mechanisms are adjusted to the gravitational field of the earth. The stability of earths gravitational field excluded evolutional adaption of biological processes to hyper- or microgravity. Gravitational changes can be associated with extreme physical forces acting on the whole organism. Rodents such as mice and rats can tolerate up to 7 g before death [1]. Therefore, extreme gravitational alteration can be considered as gravitational stress, which may have substantial adverse effects on physiological processes ranging from cellular and molecular dysfunctions to impaired tissue, organ, and immune functionalities. Space flights impose several changes to the experienced gravitational field, from hypergravity during takeoff and landing to near zero gravity in orbit. In fact, during short- and long-term space flights, functional immune dysregulation has been described in participating individuals [2, 3].

Additionally, certain physical exercises and extreme sports—including various amusement rides, skydiving, bungee jumping, or wingsuit flying—also induce gravitational stress [4–6]. All of these may potentially induced short- or long-term physiological adverse effects. For example, hypergravity exposed mice and rats displayed a normal occurrence of pregnancy, but an impaired reproducibility, where only a small fraction of neonates survived [1]. Hypergravity has further been reported to result in a reduction of the mass of murine lymphoid organs such as spleen and thymus as well as the numbers of B and T cells, which could lead to immunological dysfunctions [7].

While hypergravity can easily be mimicked by centrifugation [8], modelling microgravity is more challenging. *In vitro*, microgravity modelled using a Rotary Cell Culture System (RCCS), show that microgravity supports the formation of bone resorbing osteoclast, which is in agreement with observations from astronauts participating in long-term space missions [9]. Furthermore, dendritic cells cultivated in the RCCS system show reduced functionality, supporting the multifaceted effects of microgravity [10].

To simulate microgravity, one accepted *in vivo* model is the antiorthostatic suspension [11], where the animal is subjected to a head-down tilt. Mice exposed to this suspension model showed immune dysfunctions, with impairment of the respiratory burst responses in polymorphonuclear leukocytes [11]. Furthermore, T and B cells showed an reduced proliferative response after three weeks of hind limb unloading [12]. Naturally, animal models can only allow limited conclusions for the potential effects of gravitational stress on the human immune system.

The short-arm human centrifuge (SAHC) can be applied to investigate the effects of hypergravity on humans, and is also used to countermeasure microgravity effects during long term space flight [13, 14]. However, there are currently no studies available, which used SAHC to investigate hypergravity effects on the human immune system.

An accepted analog to study microgravity in humans is the long-duration head-down tilt bed rest [15]. After 21 days of head-down bed rest, a fraction of study participants showed a decreased cytokine responses in blood stimulated with phytohemagglutinin, indicating an impaired reactivity of certain leukocytes [16].

Space flight is obviously the most appropriate way to study long-term microgravity effects, but remains relatively inaccessible for exploratory studies in humans. Space flight studies performed in mice and humans indicated dysfunctional immune mechanisms, which could potentially increase the risk for infections, autoimmunity, or cancer [2]. However, whether those effects were solely induced by the exposure to gravitational stress remains open, since other parameter for example increased radiation and stress levels, as well as artificial light sources, could also contribute to impaired immune functions.

Parabolic flights are in this context an attractive alternative to fundamentally investigate physiological alterations including immunological functions in response to short-term gravitational stress [17]. During parabolic flights, the body is alternately exposed to phases of hyper- and microgravity. This makes it possible to evaluate how physiological processes can be influenced by gravitational stress. In fact, it could recently been shown that parabolic maneuvers led to an increase of the microbicidal function of human of polymorphonuclear leukocytes [18].

Based on the diverse observations regarding gravitational stress induced immunological alterations, we hypothesized that short-term gravitational stress can impact the number of circulating leukocyte subsets in the human blood. Accordingly, blood from 18 volunteers was analyzed immediately prior to the parabolic flight maneuvers and immediately thereafter.

Multi-parameter flow cytometry allows for in-depth phenotypic *ex vivo* characterization of circulating blood cells. Taking advantage of this technology, we performed immune cells profiling in healthy volunteers before and after parabolic flights.

Since the number of circulating leukocytes can vary over a 24 hour period [19], the circadian rhythm has to be considered when experimental observations follow a defined period [20]. Therefore, changes occurring during the parabolic flight period were compared to circadian-mediated changes occurring during the corresponding period one day before the parabolic flight. This comparison is crucial for a valid interpretation of the data, since immune mechanisms underlie bioperiodicity [21]. An aspect that was so far rarely considered in similar studies. The circadian rhythm of circulating leukocyte subsets is thought to be controlled by cortisol [22], which can also be induced in response to stress events such as parabolic flights. Therefore, cortisol levels were determined and correlated with the observed alterations in circulating leukocyte numbers. The present study demonstrates that parabolic flights can be used to investigate immunological alterations induced by gravitational stress, showing alterations in the innate and adaptive immune system.

## Materials and methods

### Study population and ethics statement

According to the criteria of the Nordkem-workshop [23], blood was obtained from 20 apparently healthy volunteers. However, one female and one male had to be excluded from the analysis since blood drawing could not be conducted during the flight (Table 1).

**Table 1:** Participant overview.

	Female	Male
**Number of participants**	8	10
**Average Age (years)**	24	28.3
**Age range (years)**	19–33	21–43

The study protocol received an approval by the ethical committee of the Ruhr-Universität Bochum (register-number 5158–14). Intake of any kind of medication on a regular basis and a history of cardiovascular diseases including hypertension, coronary heart disease, congestive heart failure, stroke, as well as immune disorders, including diabetes, asthma, or allergies were defined as exclusion criterion.

All participants provided written informed consent prior inclusion in the study. Participants of parabolic flights usually receive scopolamine as kinetosis prophylaxis. However, in order to avoid any drug-related immunological effects, we refrained from any medication. Three female volunteers showed signs of motion sickness. Each test subject was equipped with an intravenous cannula, from which the blood samples could be obtained during the flight.

### Parabolic flight and blood donation

**Fig 1:**
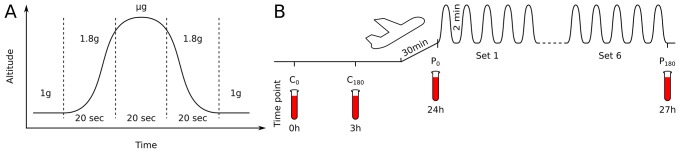
Course of a single parabolic maneuver and study scheme of immune cell analysis in human blood gravitational stress. (A) During a parabolic flight maneuver, gravitational stress appears during a 20 sec 1.8 g phase followed by a 20 sec μg phase and another 20 sec 1.8 g phase. (B) Blood was taken from 18 participants the day before the parabolic flight maneuvers (C0 and C180) and during the flight—immediately before the 31-parabolic flight maneuvers were started (P0) and immediately thereafter (P180).

An Airbus A310 performed a single successful test parabola and 30 regular parabolas in which phases of weightlessness were achieved. This microgravity phase of each parabola was flanked by a pull-up and a pull-out phase at about 1.8 g hypergravity (Fig 1A). Each phase lasted about 20 seconds. The parabolas were performed in six sets of five parabolas. Each set was separated by steady flight of 5 to 8 minutes. The steady flight in 1 g between two parabolas lasted for 2 min. The cabin conditions remained stable at all times with a pressure of 830 mbar, cabin luminosity of 800 lux and a temperature of 19–21 °C with a humidity of 15%.

Blood samples were drawn circa 30 min after takeoff prior to the first parabolic maneuver (P0) and three hours later after the last parabola (P180) before the landing procedure was initiated (Fig 1B). As control, blood was taken at corresponding time points on the ground 24 h before the parabolic flight (C0 and C180). Thus, the time span from C0 to C180 is referred to as control period and P0 to P180 as parabolic flight period (Fig 1B).

### Determination of leukocyte and leukocyte subpopulation numbers

Whole blood was processed within four hours upon donation. The immune status was evaluated using the DuraClone IM Phenotyping Basic Panel, DuraClone IM B cell Panel, DuraClone IM T cell Subset Panel, and DuraClone IM Dendritic Cell Panel (all from Beckman Coulter, Krefeld, Germany) according to manufacturer’s instructions and as illustrated in S1 Fig. Table 2 provides details on the antibody composition of the respective tubes.

**Table 2:** Antibodies of the used DuraClone panels.

DuraClone IM Panel	Antibody target	Clone	Fluorochrome
Phenotyping basic	CD16	3G8	FITC
	CD56	N901	PE
	CD19	J3-119	ECD
	CD14	RMO52	PC7
	CD4	13B8.2	APC
	CD8	B9.11	A700
	CD3	UCHT-1	APC-A750
	CD45	J33	Krome Orange
B cells	IgD	IA6-2	FITC
	CD21	BL13	PE
	CD19	J3-119	ECD
	CD27	1A4CD27	PC7
	CD24	ALB9	APC
	CD38	LS198-4-3	APC-A750
	IgM	SA-DA4	Pacific Blue
	CD45	J33	Krome Orange
T cell subsets	CD45RA	2H4	FITC
	CD197 (CCR7)	G043H7	PE
	CD28	CD28.2	ECD
	CD279 (PD1)	PD1.3.5	PC5.5
	CD27	1A4.CD27	PC7
	CD4	13B8.2	APC
	CD8	B9.11	A700
	CD3	UCHT-1	APC-A750
	CD57	NC1	Pacific Blue
	CD45	J33	Krome Orange
Dendritic cell	CD16	3G8	FITC
	CD1c	L161	PC5.5
	CD11c	BU15	PC7
	Clec 9A	8F9	APC
	CD123	107D2	APC-A700
	HLA-DR	IMMU-357	Pacific Blue
	CD45	J33	Krome Orange
Dendritic cell—lineage	CD3	UCHT-1	PE
	CD14	RMO52	PE
	CD19	J3-119	PE
	CD20	HRC20	PE
	CD56	N901	PE

Briefly, all incubation steps were performed at room temperature for 15 minutes in the dark, and all centrifugation steps were performed at 200 g, for 5 minutes at room temperature. Erythrocytes were lysed using 2 ml VersaLyse (Beckman Coulter) per reaction tube. Washing steps were performed with PBS (Fisher Scientific, Schwerte, Germany) and all samples, except the DuraClone IM Phenotyping Basic Panel, were fixed with 0.1% IOTest 3 Fixative Solution (Beckman Coulter) before analysis on a Navios flow cytometer (Beckman Coulter). The DuraClone IM Phenotyping Basic Panel and DuraClone IM B cell Panel were analyzed on the day of sample acquisition, while the DuraClone IM T cell Subset Panel, and DuraClone IM Dendritic Cell Panel were left overnight at 4°C after fixation and analyzed the following day. Cell counts were determined by adding Flow-Count Fluorospheres (Beckman Coulter) to the DuraClone IM Phenotyping Basic Panel before analysis.

Stability of the flow cytometer was assured through a daily quality control procedure using Flow-Check Pro Fluorospheres (Beckman Coulter). A compensation matrix for each panel was created using the compensation tubes supplied with each panel, according to manufacturer’s instructions.

Cortisol concentrations were measured by a solid-phase chemiluminescence immunoassay using the Immulite XPI (Siemens Healthcare Diagnostics GmbH, Eschborn, Germany).

### Statistical analysis and graphical representation

For graphical representation and statistical analysis GraphPad Prism version 7.00 (GraphPad Software, La Jolla California USA) was used. Boxes in the figures represent the 25th, 50th, and 75th percentile and the whiskers range from minimum to maximum value. The individual values are shown as points superimposed onto the boxplots. Statistical comparison using a repeated measures One Way ANOVA followed by a Sidak’s multiple comparison test was performed when more than two groups were plotted. For the statistical comparison of the C180-C0 versus P180-P0 differences, a repeated measures two-tailed t-test was applied.

## Results

### Experimental set up

During the parabolic maneuvers, the body is exposed to gravitational levels that substantially differ from that on the ground. The flight maneuver produces a number of parabolas with a phase of weightlessness at nearly 0g. This μg phase is pre- and succeeded by a hypergravity phase, with a gravitational acceleration of 1.8g (Fig 1A). To investigate potential effects of gravitational changes on major immune cells subsets and their subpopulations, blood was taken immediately prior to the parabolic maneuvers and immediately thereafter (Fig 1B). To control for possible circadian effects, blood was drawn one day before on a ground at the corresponding time points (Fig 1B).

The major leukocyte populations in whole human blood were identified through multiparametric flow cytometry (S2 Fig). A clear CD45 positive leukocyte population was ensured by doublet exclusion and flow stability verification using the time parameter followed by exclusion of unlysed erythrocytes and debris. At the same time, granulocytes can be discriminated from whole lymphocytes according to the CD45 expression level and sideward scatter (SSC) properties.

Next, among CD45^+^ granulocytes, CD16 expression was used to separate neutrophils from all other leukocytes and eosinophils, which were defined as CD16^−^ with high SSC properties (S2 Fig). The whole T cell population was identified by CD3 expression and further separated into CD4 and CD8 T cells, respectively. Natural killer (NK) cells are defined as CD56^+^ and NKT cells as CD3^+^CD56^+^ double positive. The NK cells can be further discriminated according to their CD16 expression into mature and immature NK cells (S2 Fig). B cells were identified as negative for CD3 and CD56, while positive for CD19. Monocytes and their subsets were discriminated according to CD14 and CD16 expression (S2 Fig). The basophils were identified as population with low SSC and low CD45 expression among the CD14/CD16 double negative population (S2 Fig).

### Leukocyte numbers were not substantially altered after parabolic stress

**Fig 2:**
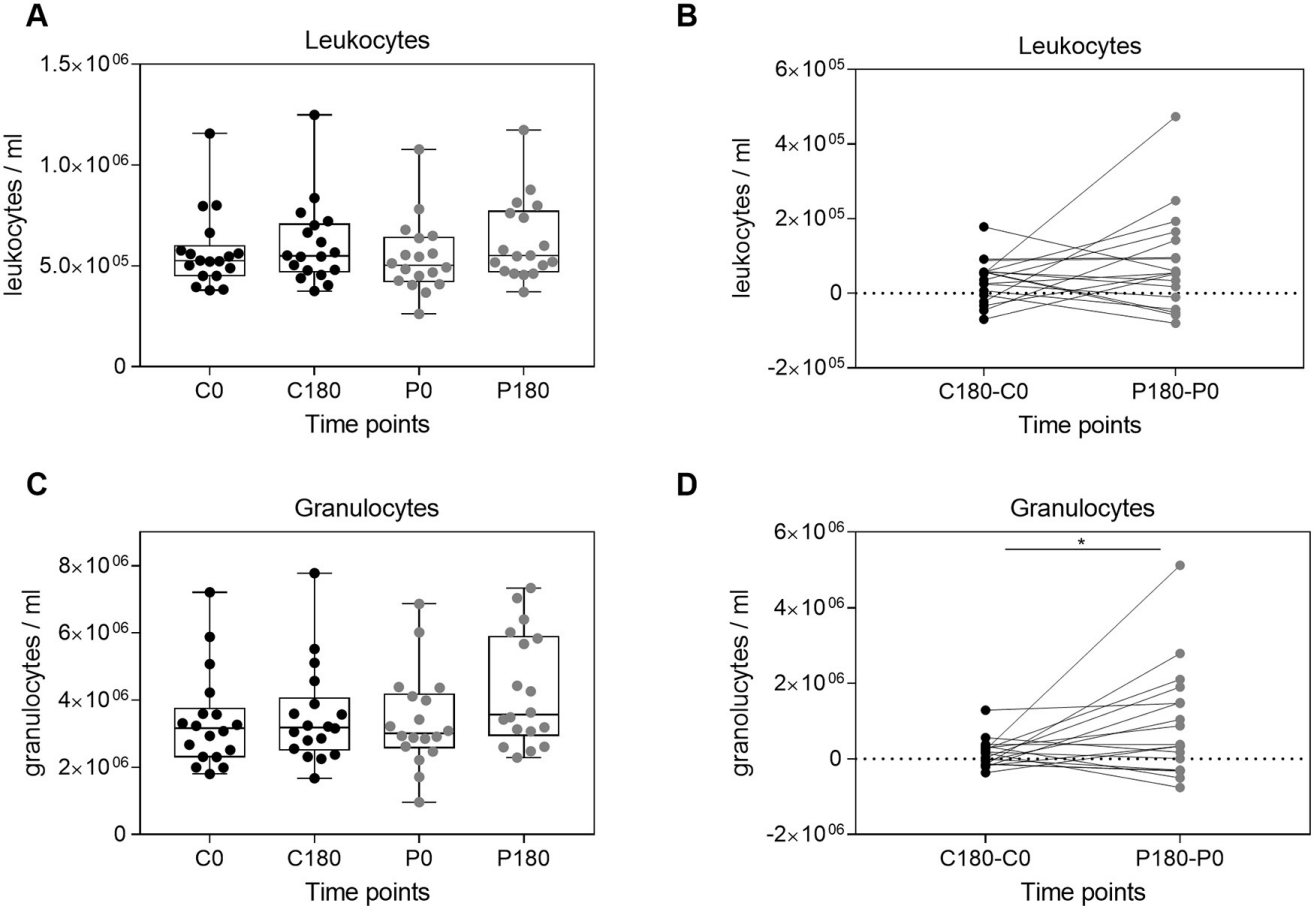
The number of circulating leukocytes remained stable, while granulocytes increased during parabolic maneuvers. Total numbers of indicated cell populations were identified by flow cytometry. Number of leukocytes (A) and granulocytes (C) shown for individual donors from the control period, one day before flight (C0) and three hours later (C180), as wells as during the flight before the parabolic maneuvers started (P0) and immediately after the 31 microgravity phases (P180). The boxes represent the 25th, 50th, and 75th percentile and the whiskers represent the range of the observations. (B) Comparison of the cell kinetics at the control period (C180-C0) with the parabolic flight period (P180-P0) for leukocytes and (D) granulocytes. Each point signifies a single donor. Asterisks indicate p-values (*** p < 0.001; ** p < 0.01; * p < 0.05) for statistical comparison.

Almost all *in vivo* processes including immunological mechanisms underlie circadian regulations [24, 25]. However, during the three-hour control period, changes in whole leukocyte counts could not be observed (Fig 2A). Similarly, the number of leukocytes circulating in the blood remained stable after exposure to gravitational changes during the parabolic flight period (Fig 2A). To clearly dissect, whether the leucocyte numbers are affected by the parabolic maneuvers or sources of variation such as the daily circadian rhythm or the difference in environmental factors, the four datasets C0, C180, P0, and P180 were normalized. Therefore, for each individual donor, C180 was subtracted from C0 (C180-C0) and P180 was subtracted from P0 (P180-P0). Subsequently, comparing the resulting difference between the control period (C180-C0) and the parabolic flight period (P180-P0) indicated that at least for a fraction of volunteers, the number of circulating leukocytes increased during the parabolic flight period. This observation could not be attributed to motion sickness observed in three volunteers (Fig 2B).

### Granulocyte numbers increased upon parabolic flight

Granulocytes compose about 60% of all leukocytes and represent the biggest white blood cells population in the human blood [26]. Therefore, it was reasonable to ask, whether also granulocytes numbers remain stable or were altered by gravitational stress, which could explain the leukocyte increase observed in a fraction of analyzed individuals. Indeed, granulocyte numbers remained stable during the control period, but showed, although not significant, a substantial increase during the parabolic flight period (Fig 2C). The comparison of the granulocyte kinetics at the control day (C180-C0 difference) with the kinetics of parabolic flights (P180-P0 difference) confirmed that the granulocyte number increased for most volunteers (Fig 2D).

**Fig 3:**
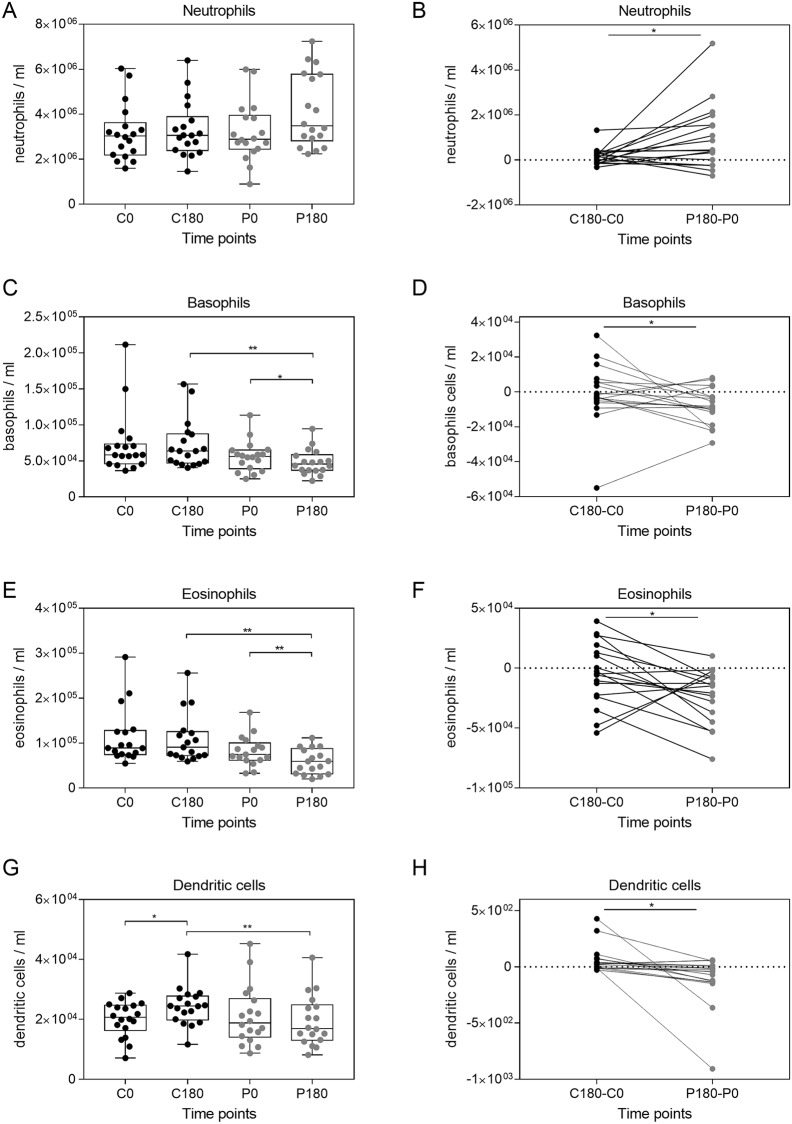
Major innate leukocyte populations are altered by parabolic flight maneuvers. The innate leukocyte populations were identified by flow cytometry and cell counts are shown for granulocytes (A), basophils (C), eosinophils (E), and dendritic cells (G). The differences between the control period (C180-C0) and the parabolic flight period (P180-P0) were compared for granulocytes (B), basophils (D), eosinophils (F), and dendritic cells (H). Asterisks indicate p-values (*** p < 0.001; ** p < 0.01; * p < 0.05) for statistical comparison. See S3 Fig for the gating strategy identifying dendritic cells.

Granulocytes is a collective term for the granular basophil, eosinophil, and neutrophil cell populations (S2 Fig). These cells respond rapidly to pathogen associated molecular pattern, allergens, and cellular debris in a non-specific fashion. Activated granulocytes can recruit other immune cells to the site of infection, induce the complement system, or release soluble inflammatory mediators [26]. We therefore evaluated the influence of gravitational stress on the cell count within each particular granulocyte population. Neutrophils are largest cell subset within the granulocytes and showed a similar kinetic pattern as was observed for the entire granulocyte population; the number of neutrophils only slightly increased due to gravitational stress (Fig 3A). Comparing cell kinetics upon parabolic flights (P180-P0) with their circadian controls (C180-C0) showed indeed a significant increase in neutrophil numbers (Fig 3B).

In contrast to neutrophils, we observed a significant decrease in the number of basophils and eosinophils during parabolic flight as compared to the control (Fig 3C and 3E). This trend could be confirmed by comparing the difference between C180-C0 and P180-P0 for basophils and eosinophils (Fig 3D and 3F).

Dendritic cells (DC) bridge the innate and adaptive immune system and can initiate and modulate T cell responses by antigen presentation and cytokine secretion [27]. DCs are predominantly resident in lymphoid tissues and organs and only low numbers can be found in the circulation. Nevertheless, circulating DCs can be quantified by flow cytometry (S3 Fig). The number of DCs significantly increased during the control period, whereas their numbers remained stable when the volunteers were exposed to gravitational stress (Fig 3G). Accordingly, we observed a significant difference in the change of DC numbers parabolic flight period in comparison to the control period (Fig 3H).

Monocytes, NKT cells, as wells as mature and immature NK cells showed no alteration, which can be attributed to gravitational stress (S4 Fig). Conclusively, our data demonstrate that numbers of granulocytes, basophils, eosinophils, and DCs change as an effect of gravitational stress.

### Gravitational stress induced a decrease in T and B cell numbers

**Fig 4:**
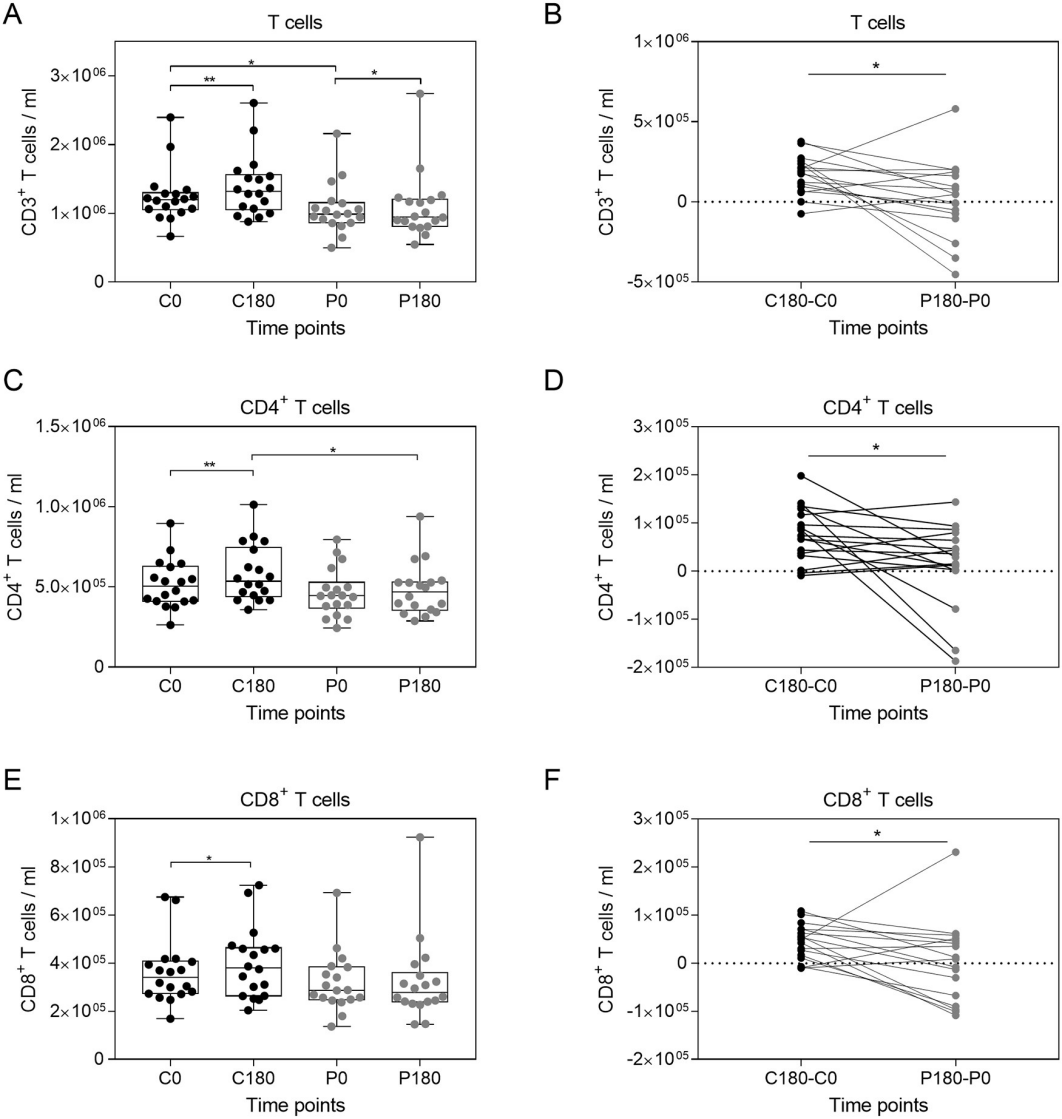
T cells numbers are altered during parabolic flight maneuvers. Total T cells were quantified by flow cytometry according to CD3 expression (A). The CD3 positive T cells were further discriminated into CD4 (C) and CD8 (E) expressing T cells, respectively. The differences between the control period and the parabolic flight period were compared for the whole CD3^+^ T cell pool (B), CD4^+^ T cells (D), and CD8^+^ T cells (F). Asterisks indicate p-values (** p < 0.01; * p < 0.05). See S5 Fig for details on the gating strategy.

Formation of immunological memory and the generation of specific protective antibodies is the hallmark of the adaptive immune system. The major cell populations of the adaptive immune system consist of T cells, which can be identified by CD3 expression, and of B cells identifiable by CD19 expression [28]. The CD3^+^ T cell population can be classified as CD4^+^ T cells and CD8^+^ T cells. To analyze whether the whole T cell pool or specific T cell subpopulations, including memory subsets, are more susceptible to gravitational stress, the T cell populations were quantified by flow cytometry (S5 Fig). Interestingly, the number of T cells increased during the control period, but decreased significantly after the parabolic maneuvers (Fig 4A). This decline became even more evident, when the changes in T cell numbers between the control period and the parabolic flight period (P180-P0 vs. C180-C0) were compared (Fig 4B). A similar effect could be observed for the CD4 and CD8 positive T cell population (Fig 4C and 4E). However, comparison of C180-C0 and P180-P0 did not show significant differences, but highlights that at least for a fraction of volunteers, the T cell numbers including CD4^+^ and CD8^+^ dropped substantially (Fig 4B, 4D and 4F).

Next, we asked whether the differences in CD4^+^ and CD8^+^ T cells numbers induced by gravitational changes could be explained by changes in distinct memory cell populations or if all populations were equally affected (S6 Fig). While the number of total CD4^+^ T cells was influenced by gravitational stress, significant alterations could not be observed for distinct CD4^+^ T cell subsets such as naïve CD4^+^ T cells, CD4^+^ effector memory T cells (TEM), CD4^+^ central memory T cells (TCM), and the subset of T effector memory, re‐expressing CD45RA (TEMRA) (S6 Fig).

**Fig 5:**
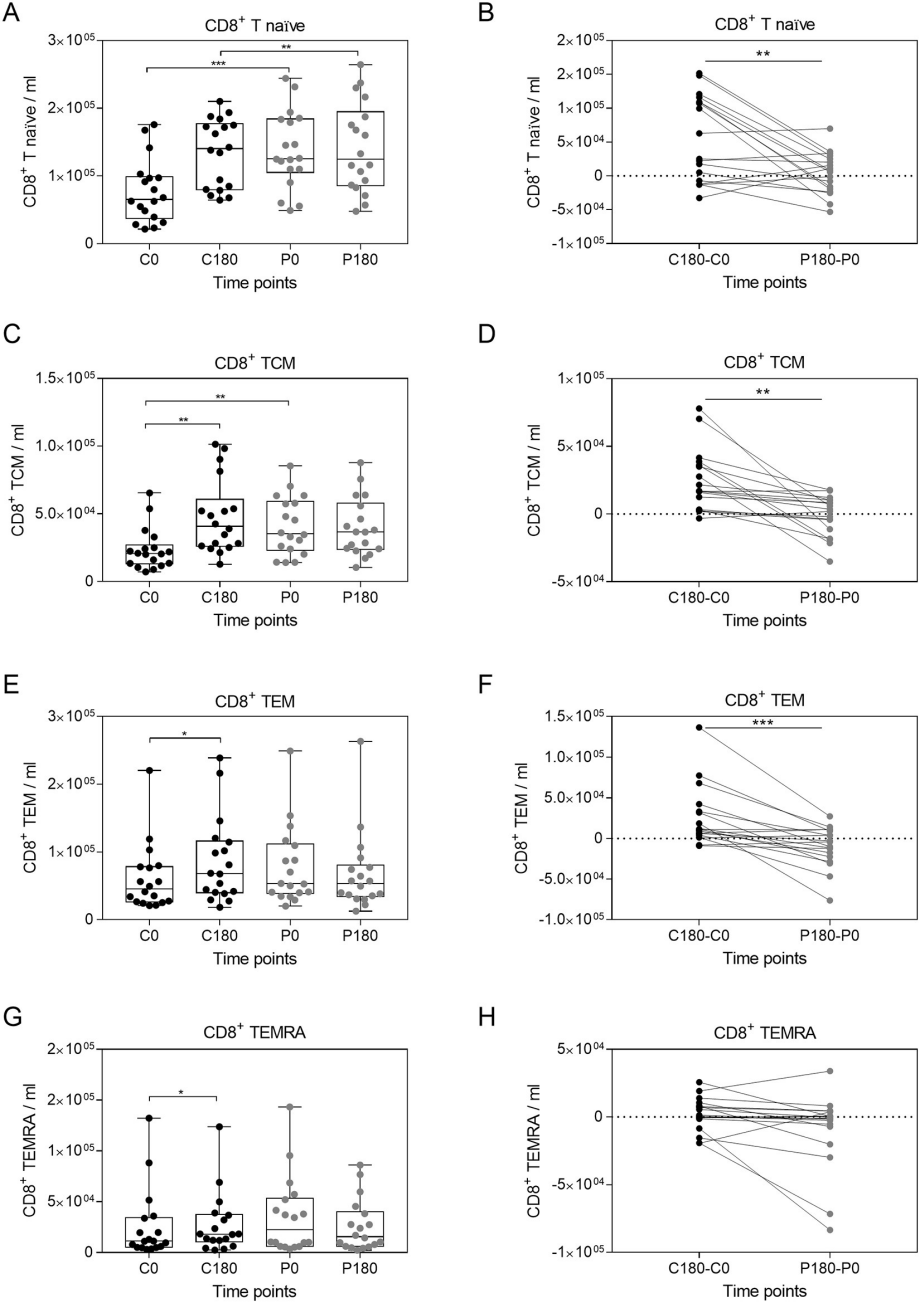
Naïve and memory CD8^+^ T cells are sensitive to gravitational stress. CD8 positive memory and naïve T cells were discriminated according to CD45RA and CCR7 expression (S5 Fig). Quantification of naïve CD8^+^ T cells (A) CD8^+^ TCM (C) and CD8^+^ TEM (E) cell number. The differences between the control period and the parabolic flight period were compared for naïve CD8^+^ T cells (B) CD8^+^ TCM (D) and CD8^+^ TEM (F). Asterisks indicate p-values (***p < 0.01; ** p < 0.01; * p < 0.05) of the statistical comparison.

In contrast, the number of CD8^+^ populations including naïve CD8^+^ T cells, CD8^+^ TCM, and CD8^+^ TEM increased during the control period; whereas this circadian rhythm mediated increase was abolished by gravitational stress (Fig 5A, 5C and 5E). Accordingly, comparing the differences between the control period (C180-C0) and the parabolic flight period (P180-P0) we found a significant drop of CD8^+^ T cells, CD8^+^ TCM, and CD8^+^ TEM (Fig 5B, 5D and 5F). Conclusively, the observed change in T cell populations shows that the cellular composition of the adaptive immune system is affected by gravitational stress.

**Fig 6:**
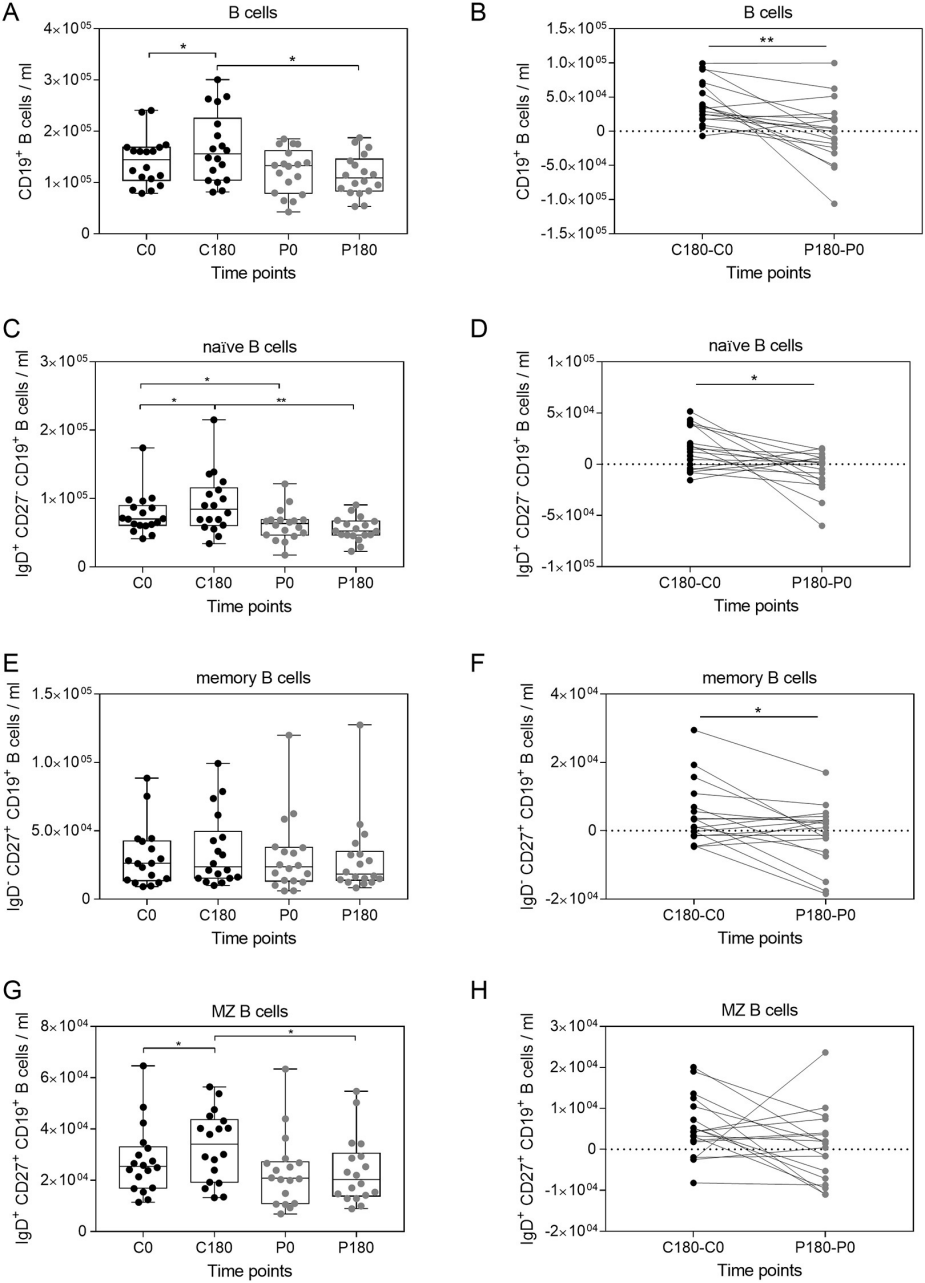
Circulating B cell populations can be influenced by gravitational stress. B cells were identified according to CD19 expression among the CD45^+^ lymphocyte population by flow cytometry (S7 Fig). Naïve (C), memory (E), and marginal zone like (MZ) B cells (G) were identified and quantified according to IgD and CD27 expression. The differences between the control period and the parabolic flight period are shown for naïve B cells (B) memory B cells (D) and MZ B cells (F). Asterisks indicate p-values (***p < 0.01; **p < 0.01; *p < 0.05) of the statistical comparison.

B cells play a major role in establishing protective long-term immunity by secretion of antibodies and initiation of T cell responses by presentation of antigens. Additionally, B cells can regulate immune reactions by the secretion of cytokines [29]. Similar to the whole T cell population the total number of B cells significantly increased during the three hours control period, while this increase was abolished after exposure to gravitational stress (Fig 6A).

The blood circulating B cell population can be classified into naïve B cells and memory B cells according to their expression profiles of CD27 and IgD (S7 Fig). IgD^+^CD27^−^ B cells are considered as naïve B cells and IgD^−^CD27^+^ are class-switched memory B cells [30]. The IgD^+^CD27^+^ double positive population refers to circulating marginal zone-like B cells (MZ) including a substantial amount of non-switched memory B cells (S7 Fig) [31]. Naïve and MZ B cells show a similar susceptibility to gravitational stress, since their numbers increased during the control period, but remained at stable levels when the body was exposed to gravitational stress. The number of circulating memory B cells was not affected by gravitational stress (Fig 6C, 6E and 6G). The gravitational-induced reduction of B cells could be demonstrated for naïve and memory B cells by comparing the differences between the control period and the parabolic flight period. For MZ B cells this trend is only apparent for a fraction of volunteers (Fig 6D, 6F and 6H). Conclusively, both T cells and B cells, as major adaptive immune cell populations, demonstrate a similar response pattern to gravitational stress, in which their normal circadian increase is abolished after exposure to gravitational changes.

### Cortisol did not explain the observed alterations in circulating leukocyte populations

Cortisol has been described as co-regulator molecule controlling the circadian rhythm of circulating leukocytes [32]. It exhibits a robust time-of-day-dependent rhythm in humans and is assumed to usually peak in the morning. However, cortisol levels can change in stress situations [22]. Given that parabolic flight is a highly unusual situation, we hypothesized that the observed alterations of leukocyte populations is ascribed to stress induced changes in blood cortisol levels.

**Fig 7:**
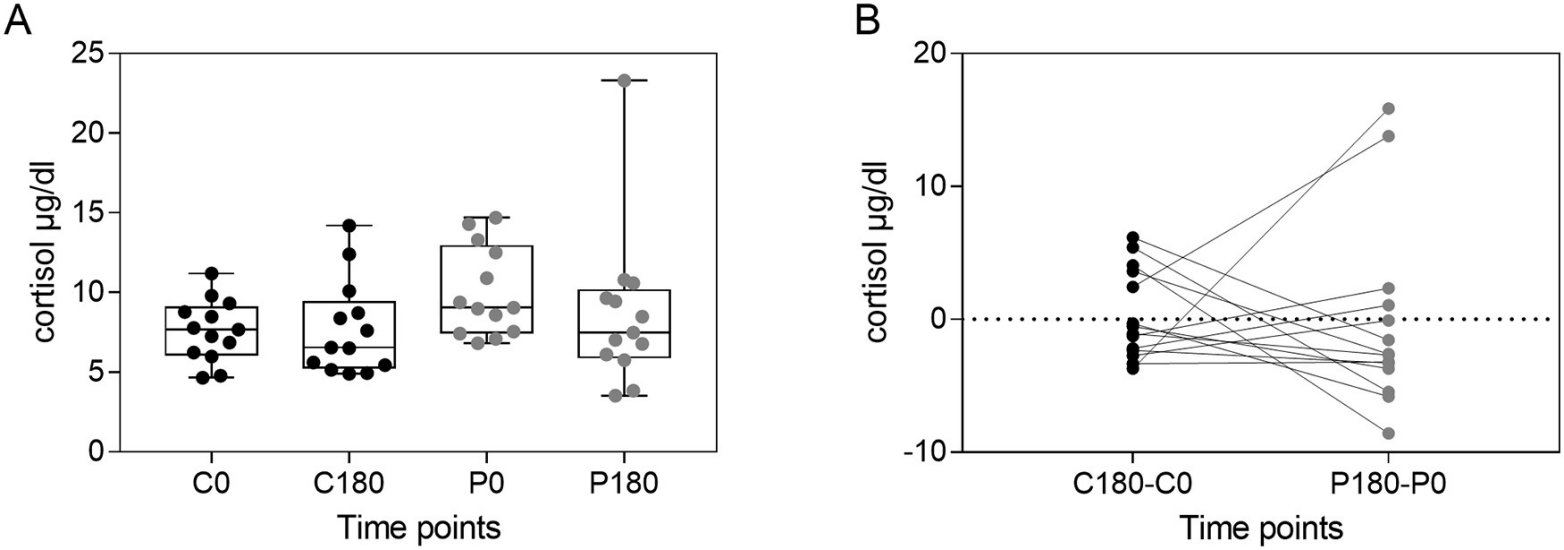
Cortisol levels are not influenced by parabolic flight maneuvers. Cortisol was determined in blood taken at control (C0, C180) and flight time points (P0, P180). (A) Cortisol levels of the individual donors at the different time points. (B) Comparison of the difference between control period and parabolic flight period.

The parabolic flight took place at 8 am, accordingly cortisol levels were determined at 8 am and 11 am for both the control period and the parabolic flight period. Interestingly, no significant circadian increase was observed for the control period (Fig 7A). At the flight day, the initial cortisol levels (P0) were slightly, albeit insignificantly, increased compared to the control time point (C0). However, comparison of the post flight value with the corresponding control value showed a normalization of the cortisol values after the flight (Fig 7A). Furthermore, comparison of the cortisol changes during the control period with the parabolic flight period revealed no significant differences (Fig 7B). Collectively, we conclude that changes in cortisol levels are not explanatory for the observed gravitational stress induced alterations of leukocyte populations.

## Discussion

Gravitational stress can be experienced not only during space flights, but also through several earthbound activities such as certain amusement activities or extreme sports. As with stress in general, gravitational stress can potentially influence immunological mechanisms [33]. Long-term exposure of astronauts to microgravity, cell culture experiments, as well as altered gravity models for humans and mice could indeed confirm impaired immune responses after exposure to gravitational stress [10, 12, 13, 16, 34]. However, the exact mechanism of how gravitational stress influences the immune system are completely unclear. Furthermore, it needs to be considered that space flight data were obtained from a very limited number of individuals, all of which were pre-selected according to high psychological and physiological fitness [35].

Although, participation in parabolic flights also requires a certain physiological fitness, the requirements for participation are less restricted compared to space flights, SAHC, and long-term bed rest studies. Thus, parabolic flights are qualified as suitable short-term model to investigate gravity induced physiological changes in diverse pools of individuals. In this initial study only apparently healthy individuals participate in the parabolic flight, in order to proof whether parabolic flights are feasible for investigating gravitational effects on the immune system. Blood from 18 volunteers was analyzed for alterations in circulating leukocyte subsets after gravitational stress. Granulocytes and neutrophils showed increasing cell numbers upon parabolic maneuvers. In contrast, basophils and eosinophils, as well as T cells and B cells including their respective subpopulations showed decreased cell numbers. Thus, our results show that gravitational stress has differential effects on leukocyte populations.

The observed drop of T cell numbers in peripheral circulation is in agreement with the data obtained from space flights, bungee jumping studies, and hindlimb unloading of mice [6, 12, 35]. The decrease of circulating T and B cells might be explained by an increased apoptosis rate, which has been demonstrated for Jurkat cells, a human T cell line, exposed to microgravity [36]. Another reason for the declining lymphocyte numbers could be their migration into lymphoid organs, as suggested by studies performed in rats, showing that CD4^+^ and CD8^+^ T cells numbers increased in the spleen during space flight [37]. While a 45 days head-down bed rest study showed a decrease in activated and memory-like tissue B cells [38], a space flight-induced increase in CD19^+^ B cells could be attributed to increases in resting memory B cells [39]. Our data show a decrease of B cells and B cell subpopulations after parabolic maneuvers, which indicates that parabolic as well as space flights induce different effects in B cells.

In fact, when comparing space flight data with data obtained from parabolic flights, it needs to be considered that microgravity remains constant over a distinctly longer period during space flights, while hyper- and microgravity alternate during parabolic flights. There is currently only a very limited number of ground based studies available aiming to confirm the immunological data obtained during space flight. Moreover, data obtained from bed rest studies are rather conflicting. For example, one study showed no substantial adverse effect on human immune function after 21 days of head-down tilt bed rest, while others observed an impaired reactivity of certain leukocytes in at least a fraction of individuals [16, 40].

Whether the altered leukocyte numbers induced by parabolic maneuvers is associated with functional consequences such as impaired immunity, which was suggested by space- and parabolic flight data [18, 34], remains to be confirmed. Moreover, systematic studies including *in vitro*, as well as *in vivo* studies in humans and animals are needed to facilitate gravitational stress induced immune alterations. Our data indicate that the parabolic flights is a suitable model to be included in such comprehensive studies, since specific aspects of space flights and other gravitational stress inducing activities can be mimicked. Studies with bigger cohorts could also consider other demographic parameters such as age and gender, evaluating whether gravitational changes can affect men and women differentially.

Previously, it was shown that, the number of circulating naïve CD4^+^ and CD8^+^ T cells declined with daytime progression, while effector CD8^+^ T cell counts peak during daytime [19, 22]. This circadian rhythm dependent variation was considered in our experimental setup, since the control period was established, by drawing blood one day prior to the parabolic flight at the corresponding time points. Circadian changes in cortisol levels are associated with the circadian rhythm of circulating leukocyte numbers [32]. For example, T cell counts in the blood are controlled by circadian variation in cortisol production, which regulates the expression of CXCR4 on T cells, leading to their re-migration into the bone marrow, following the gradient of CXCL12, the ligand of CXCR4 [22]. Furthermore, stress hormone induction mediated by bungee jumping led to a suppression of innate immune mechanisms [6]. These data led us assume that gravitational stress induced changes in cortisol levels can mediate the alterations in leukocyte numbers. However, significant increases of cortisol levels mediated by parabolic maneuvers could not be detected, indicating that cortisol was not responsible for the observed changes in leukocytes numbers. We did observe higher cortisol levels immediately prior to the parabolic maneuvers as compared to the corresponding control time point. An observation we attribute to an increased stress like excitement of the volunteers in anticipation of the flight.

In addition to the gravitational stress during parabolic flights, there are other environmental parameters potentially affecting the whole organism and subsequently the leukocyte number. This includes intensified radiation, increased light intensities, and sterility of the environment, which can be insufficiently simulated on earth, but have to be considered and controlled in future studies. In fact, data from a simulated long-haul flight also suggests an impairment of certain immune mechanism indicating that besides gravitational stress other environmental parameters such as hypobaric hypoxic conditions could mediate immune dysfunctions [41].

Whether the immunological alterations mediated by gravitational stress could affect the susceptibility for infections, auto-immunity, or tumors should also be analyzed in the future for example *in vitro* by stimulation of certain leukocyte subsets isolated after exposure to gravitational stress.

In conclusion, our study demonstrates that even short-term exposure to gravitational stress affects the composition of the innate and of the adaptive immune system. Repeated subjection to gravitational stress can therefore have adverse consequences for the efficacy of the immune response.

## Supporting information

S1 Fig Staining procedure. Overview of the different staining procedures to identify and quantify leukocyte populations in whole human blood by flow cytometry.
(TIFF)

S2 Fig Gating strategy. Identification of major immune cell subsets in whole human blood. The gray box indicates an exclusion gate.
(TIFF)

S3 Fig Gating strategy. Discrimination of granulocytes from lymphocytes and the identification of circulating DC.
(TIFF)

S4 Fig Cell count of innate cell populations. Cell counts for classical monocytes (A), NKT cell (C), mature NK cells (E), and immature NK cells (G). Differences between the control period (C180-C0) and the parabolic flight period (P180-P0) were compared for classical monocytes (B), NKT cells (D), mature NK cells (F), and immature NK cells (H).
(TIFF)

S5 Fig Gating strategy to identify memory populations of CD4^+^ and CD8^+^ T cells. The gray box indicates exclusion before application of the inclusion gate.
(TIFF)

S6 Fig Cell count for CD4^+^ T cells. Cell counts and differences between the control period (C180-C0) and the parabolic flight period (P180-P0) for naïve CD4^+^ T cells (A, B), CD4^+^ TEM (C, D), CD4^+^ TCM (E, F), and CD4^+^ TEMRA (G, H). Asterisks indicate p-values (***p < 0.01; **p < 0.01) of the statistical comparison.
(TIFF)

S7 Fig Gating strategy. Identification of total circulation B cells and their subpopulations. The gray box indicates exclusion before application of the inclusion gate.
(TIFF)
