# Supplementary figures and images for: Gravitational stress during parabolic flights reduces the number of circulating innate and adaptive leukocyte subsets in human blood
Source: PLoS One. 2018 Nov 14;13(11):e0206272. doi: 10.1371/journal.pone.0206272 (PMC6235284; doi:10.1371/journal.pone.0206272)

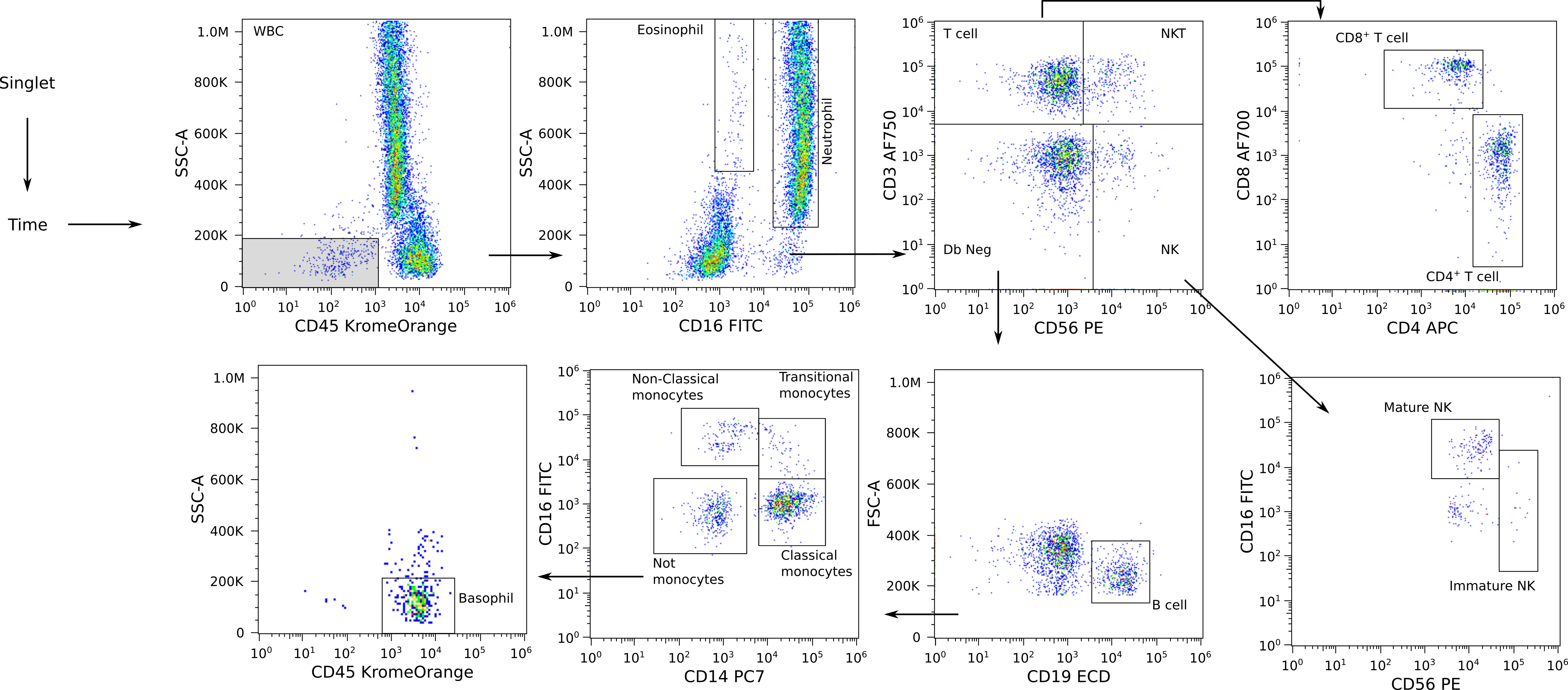

Supplement: S2 Fig — Identification of major immune cell subsets in whole human blood. The gray box indicates an exclusion gate. (TIFF) [file pone.0206272.s002.tiff]

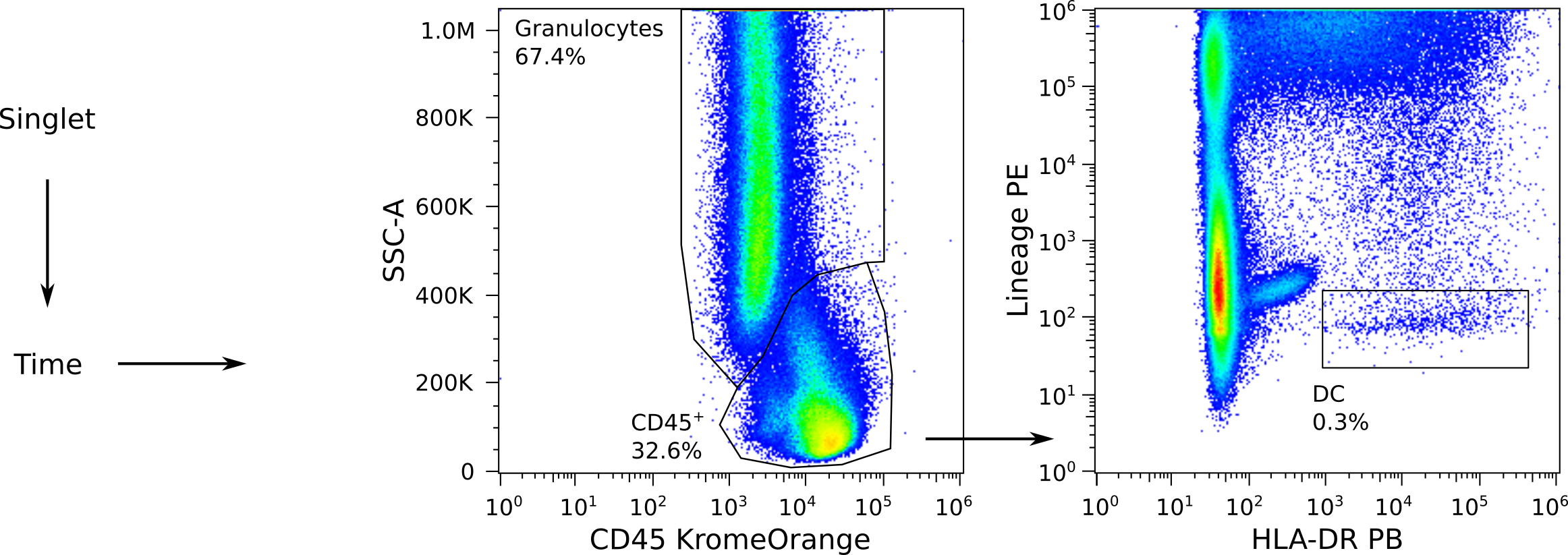

Supplement: S3 Fig — Discrimination of granulocytes from lymphocytes and the identification of circulating DC. (TIFF) [file pone.0206272.s003.tiff]

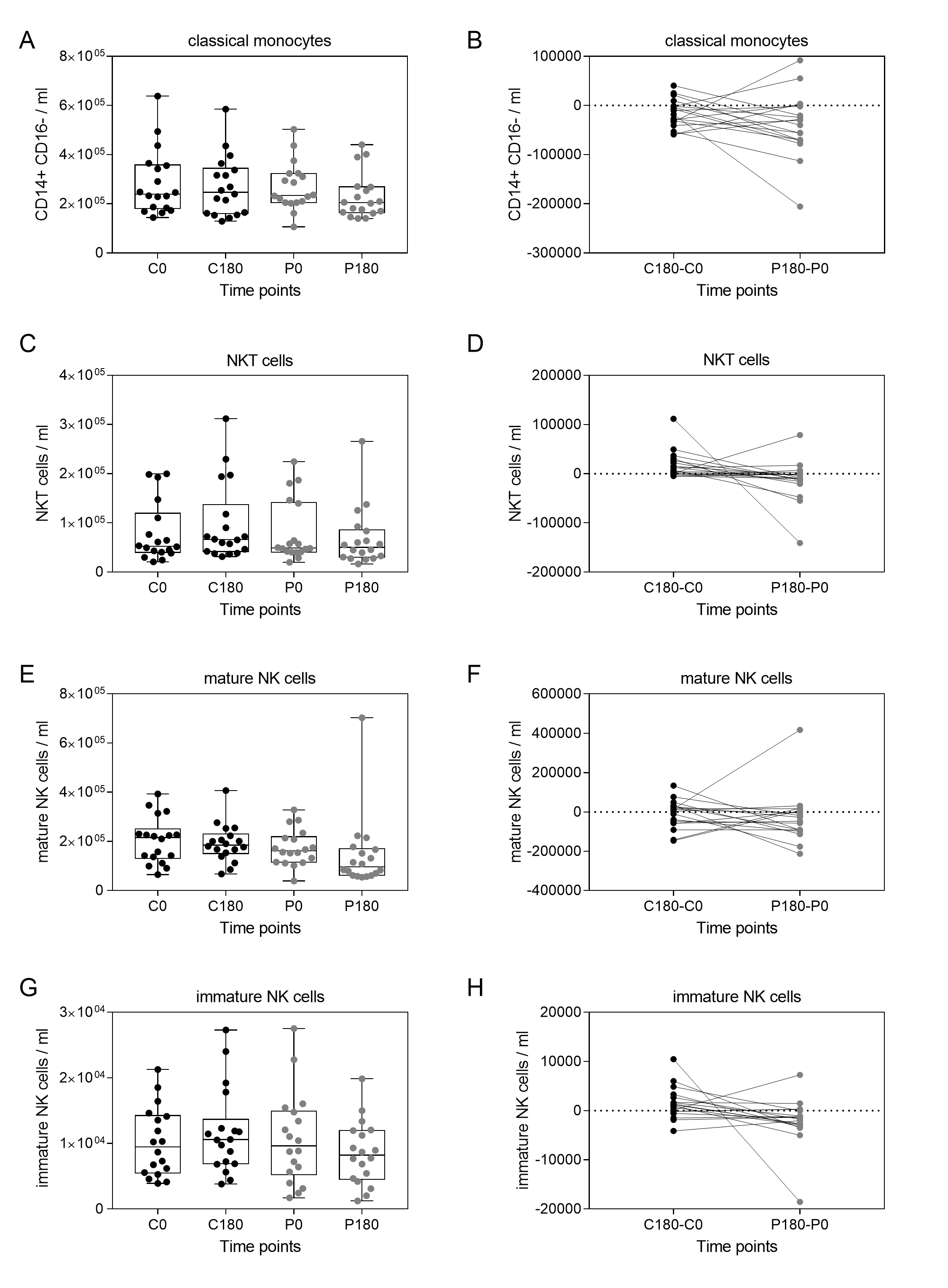

Supplement: S4 Fig — Cell counts for classical monocytes (A), NKT cell (C), mature NK cells (E), and immature NK cells (G). Differences between the control period (C180-C0) and the parabolic flight period (P180-P0) were compared for classical monocytes (B), NKT cells (D), mature NK cells (F), and immature NK cells (H). (TIFF) [file pone.0206272.s004.tiff]

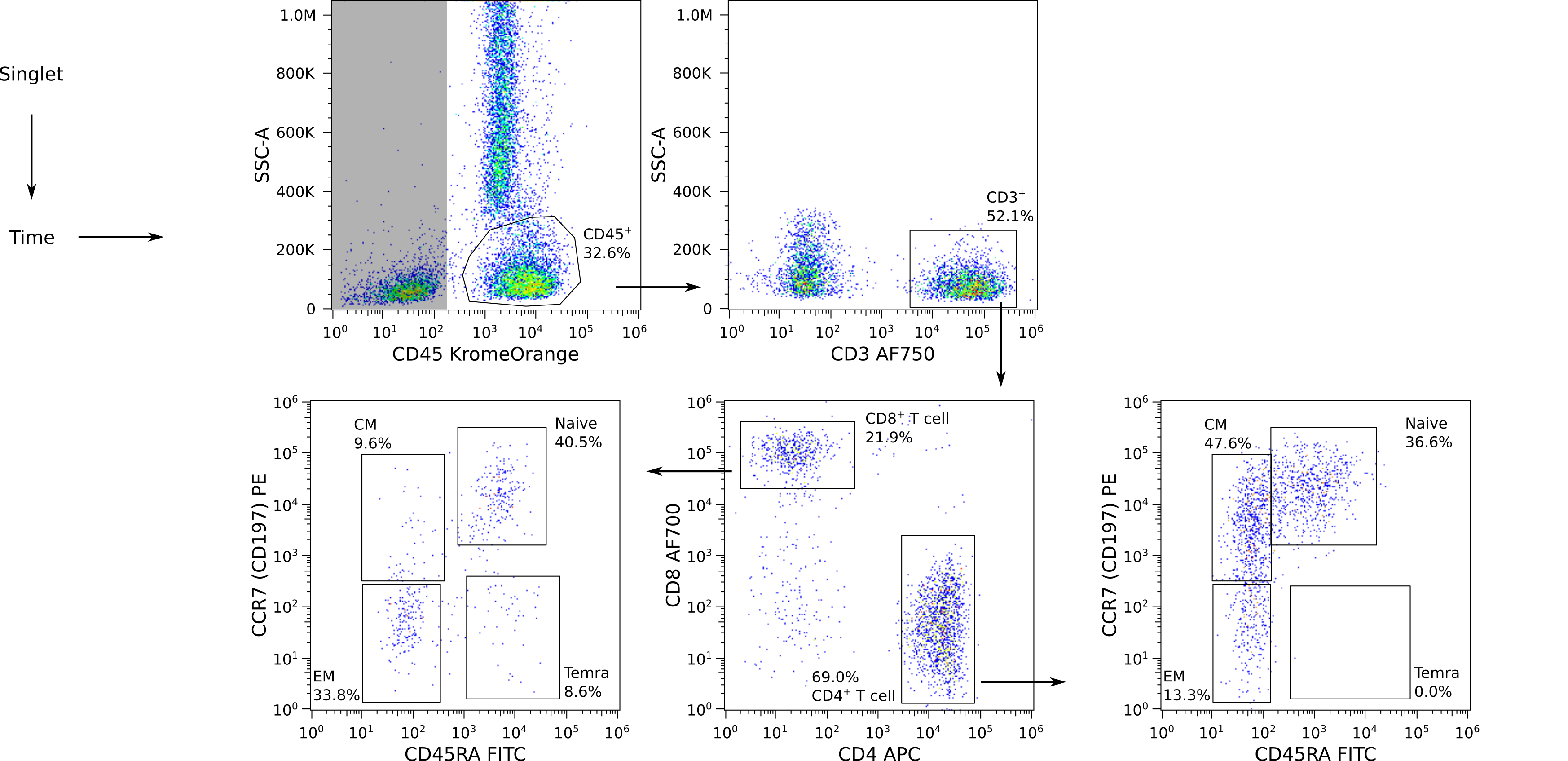

Supplement: S5 Fig — The gray box indicates exclusion before application of the inclusion gate. (TIFF) [file pone.0206272.s005.tiff]

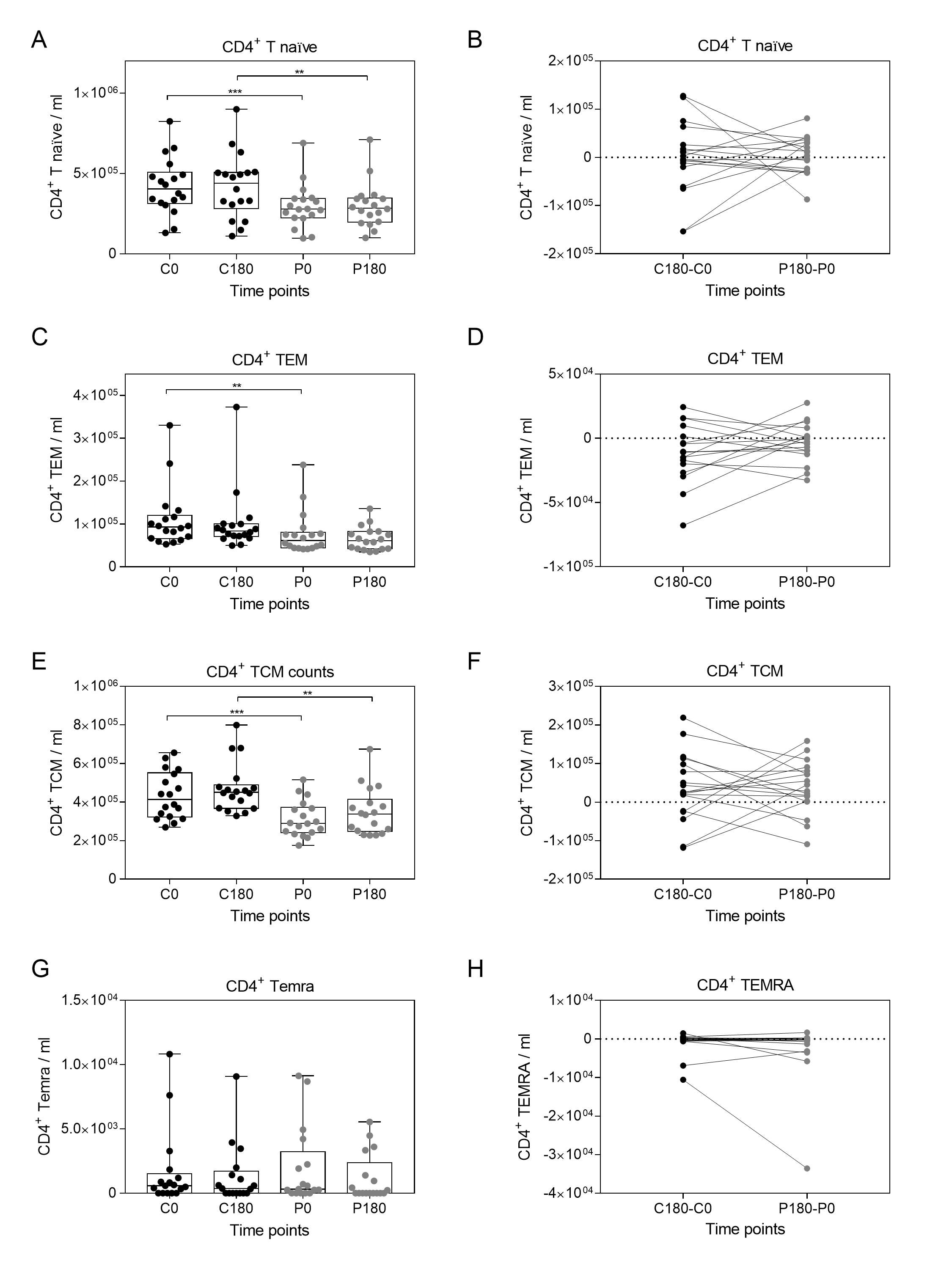

Supplement: S6 Fig — Cell counts and differences between the control period (C180-C0) and the parabolic flight period (P180-P0) for naïve CD4+ T cells (A, B), CD4+ TEM (C, D), CD4+ TCM (E, F), and CD4+ TEMRA (G, H). Asterisks indicate p-values (***p < 0.01; **p < 0.01) of the statistical comparison. (TIFF) [file pone.0206272.s006.tiff]

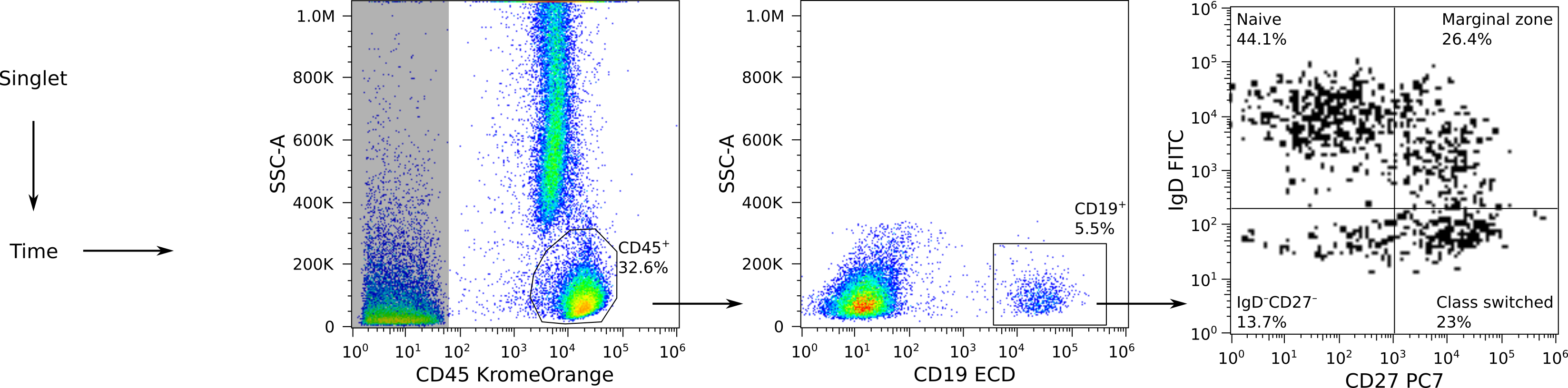

Supplement: S7 Fig — Identification of total circulation B cells and their subpopulations. The gray box indicates exclusion before application of the inclusion gate. (TIFF) [file pone.0206272.s007.tiff]
